# Early determinants of food liking among 5y-old children: a longitudinal study from the EDEN mother-child cohort

**DOI:** 10.1186/s12966-016-0342-5

**Published:** 2016-02-15

**Authors:** Wen Lun Yuan, Natalie Rigal, Sandrine Monnery-Patris, Claire Chabanet, Anne Forhan, Marie-Aline Charles, Blandine de Lauzon-Guillain, on behalf of the EDEN mother-child cohort Study Group

**Affiliations:** Centre des Sciences du Goût et de l’Alimentation (CSGA), CNRS, UMR6265, F-21000 Dijon, France; Centre des Sciences du Goût et de l’Alimentation (CSGA), INRA, UMR1324, F-21000 Dijon, France; Centre des Sciences du Goût et de l’Alimentation (CSGA), Bourgogne Franche-Comté University, F-21000 Dijon, France; Paris-Ouest University, F-92001 Nanterre, France; Early ORigin of the Child’s Health and Development Team (ORCHAD), Epidemiology and Biostatistics Sorbonne Paris Cité Center (CRESS), INSERM, UMR1153, F-75014 Paris, France; Paris-Descartes University, F-75005 Paris, France

**Keywords:** Children, Food liking, Determinants, Food neophobia, Longitudinal

## Abstract

**Background:**

Identifying the determinants of child’s liking for different foods may help to prevent future choices of unhealthy food.

**Objective:**

To study early-life food-related characteristics associated with child’s liking for different foods at 5y with a longitudinal study.

**Design:**

1142 5y- old children completed a liking test for “fruit and vegetables”, “meat, fish and eggs”, desserts and cheese. Data related to maternal food intake before pregnancy, infant feeding during the first year of life, maternal feeding practices at 2y, child’s food intake at 3y, and child’s food neophobia from 1 to 4y were collected prospectively from the mother. The associations between these factors and child‘s liking for each category of foods were analyzed using structural equation modelling.

**Results:**

High food neophobia at 4 y was related to lower child’s liking for all food groups. Maternal feeding practices at 2y were associated with liking for dessert: negatively for the practices allowing child to control his/her own food intake, positively for restriction of child’s food intake for weight reasons. Moreover, child’s food intake at 3y was positively associated with child’s liking for “fruit and vegetables” as well as for cheese. Finally, adherence to the infant feeding pattern “long breastfeeding, later introduction of main meal components and use of home-made products” was positively associated with child’s liking for meat/fish/eggs.

**Conclusions:**

For all food groups, food neophobia was a common determinant of child’s liking for food at 5y, whereas other factors were associated with food liking for specific food groups.

**Electronic supplementary material:**

The online version of this article (doi:10.1186/s12966-016-0342-5) contains supplementary material, which is available to authorized users.

## Background

Healthy and unhealthy eating habits established in early childhood appear to track into adulthood [1]. Understanding the early determinants of food liking may have practical implications to healthy eating habits since among school-age children, previous studies have shown that food intake is driven by liking the foods [2, 3]. Previous studies on the development of food liking have investigated perinatal exposures, child’s temperament especially food neophobia and parental feeding practices [3–12].

Early repeated exposure to a flavor or a food appeared to increase liking or preferences for that food in childhood [3–6]. Even exposures during the in-utero period or during lactation have been shown to have an influence on infant food liking [4]. Nevertheless, to our knowledge, few studies have examined the role of infant feeding practices during the first year of life on food liking later in childhood or in adulthood.

Although food neophobia, defined as the avoidance of and reluctance to taste unfamiliar foods [13], may lead to a liking for a narrow range of foods [9–11], its influence at the early ages on future liking for specific foods is poorly investigated. Since food neophobia was shown to be accentuated by the type of food presented, such as vegetables [10], most studies were focused on association with fruit and vegetables. Furthermore, most observational studies have shown cross-sectional associations between food neophobia and food intake [9, 11]. Consequently, the influence of food neophobia on later liking for different food groups is poorly studied, except for fruit and vegetables.

Parental influences on child’s liking for specific foods rely on their own food intake or on their child-feeding practices [12, 14–17]. Parents tend to offer the foods they like and not the ones they dislike, limiting the range of varied flavors to which the child is exposed [9]. Beyond general parenting style (authoritative, authoritarian, indulgent or neglectful) [18], there are specific food-related parenting practices which could have either negative influence on children eating behavior such as restriction for weight, pressure to eat, food as reward, emotion regulation, child control or positive one such as teaching nutrition or encouraging balance and variety [19, 20]. In experimental studies, restriction of sweet foods makes child focuses more on the restricted sweet foods than on the non-restricted ones [14]. Moreover, when these restricted foods become available, the child eats more of them than an equally liked food at baseline or eats them without being hungry [14, 15]. In contrast, a permissive feeding style implies a higher child control [21], which is a high responsiveness to child’s requests and low demands on him or her, has been shown to be associated with higher non-core food intake among children [16] and child self-serving during the meal was associated with higher energy intake [17]. In other terms, parents with a permissive feeding style tended to offer only foods that child liked or let child choose what he/she would like to eat, which are usually highly palatable such as sweet foods.

The present study aims to examine with a longitudinal study design the relative contribution of internal determinants (child’s food neophobia from 1 to 4y, child’s food intake at 3y) and external determinants (infant feeding practices, parental feeding practices at 2y, maternal food intake during pregnancy) on the child’s food liking at 5y, assessed by a face-to-face interview.

## Methods

### Subjects

Participants were subjects from the EDEN mother-child cohort. The main objective of the EDEN cohort was the study of pre-natal and early post-natal nutritional, environmental and social determinants of child’s development and health. Between 2003 and 2006, 2002 pregnant women were recruited in two French University hospitals, Nancy and Poitiers. Poitiers region is more rural than Nancy region. Exclusion criteria were twin pregnancies, known diabetes before pregnancy, moving outside the region planned within the next 3 years and not being able to speak and read French. The detailed study protocol has been published previously [22]. Written consent was obtained from the mother for herself and for the newborn at birth. The study was approved by the ethics committee (CCPPRB) of Kremlin Bicêtre and by the Data Protection Authority “Commission Nationale de l’Informatique et des Libertés” (CNIL).

### Measures

Socio-demographic data were collected by questionnaire during pregnancy, at 24–28 weeks of amenorrhea. Birth data were collected from medical records by trained midwives.

#### Maternal diet before pregnancy

During the first trimester of pregnancy, a food frequency questionnaire assessed mothers’ diet in the year before pregnancy. This questionnaire was very similar to the questionnaire validated and developed for the French population in the Fleurbaix-Laventie Ville Santé (FLVS) Study [23]. The questionnaire included 137 items each with 7 frequency categories ranging from never to more than once a day [24].

#### Infant feeding practices

At 4, 8 and 12 months of age, mothers completed mailed questionnaires with details on the feeding method and the age of introduction to several food groups. At 12 months of age, a sub-group of mothers also completed a questionnaire on the specific type of food used at this age (home-made, ready-prepared baby food, ready prepared adult food). Infant feeding patterns have previously been identified from these data (breastfeeding duration, age of introduction to 14 complementary foods, type of food used at 12 months of age above mentioned) by principal component analysis [25]. Three feeding patterns were characterized: pattern-1 labelled ‘Later dairy products introduction and use of ready-prepared baby foods’, pattern-2, labelled ‘Long breastfeeding, later main meal food introduction and use of home-made foods’ and pattern-3, labelled ‘Use of ready-prepared adult foods’, establishing 3 independent variables.

#### Parental feeding practices

At 2y of age, parental feeding practices were evaluated using the Comprehensive Feeding Practices Questionnaire (CFPQ) [21]. In the present analysis, we used the only scales related to food liking in our sample, i.e., “Restriction For Weight Control” (RFW, parents control the child’s food intake with the purpose of decreasing or maintaining the child’s weight) and “Child Control” (CC, parents allow the child to control his/her eating behaviors and parent-child feeding interactions”). Each feeding practice was assessed with three items ranging from 1 to 5.

#### Child’s diet

At 3y of age, a 26-item food frequency questionnaire concerning the child’s diet was completed by mothers. Each item includes 7 frequency categories ranging from never to more than once a day. The intake of “fruit and vegetables”, “meat, fish and eggs”, “dairy product” and desserts were assessed respectively from 4 items (raw vegetables, cooked vegetables, fresh fruit, stewed fruits), 6 items (ham, processed meat, meat, fatty fish, lean fish, eggs), 3 items (yogurt, cheese, milk) and 2 items (dairy desserts, cookies). The dairy desserts item included entremets and ice cream while the cookies item included cookies, cakes and pastries.

#### Food neophobia

Child’s food neophobia was assessed, at age 1y, using two items: “My child eats new food without difficulties” and “My child doesn’t like a lot of foods”, at ages 2, 3 and 4y, using two items “My child doesn’t like to taste new foods” and “My child refuses to taste new foods”. From 1 to 4y, answers were collected on a 4-point (1 to 4) scale ranging from “totally disagree” to “totally agree” and a food neophobia score was calculated at each follow up as the mean of both items. Items used at 1y were related to both pickiness and neophobia, whereas they were only associated with neophobia from 2 to 4y because neophobia is supposed to become predominant over the age of 2y [26].

#### Food liking assessment

Child’s liking for foods was assessed during the clinical visit at 5y of age, with a face-to-face interview conducted by a trained midwife research assistant. Parents were not present during the interview to reduce the social desirability bias. During the interview, pictures of 36 food items were shown to the child. For example, with a picture representing a pear, the question was asked: “this is a pear, can you tell me how you like it?”. They expressed their liking by moving the position of the cursor on a hedonic gradient scale with 3 smileys as references (a positive face, a neutral face and a negative face) on the front side, and a linear scale ranked from 0 to 10 on the back-side only visible for the experimenter. We performed an internal validation of this food liking measurement method in three steps. First, a random sample of the participants (*n* = 571) was used to build a Confirmatory Factor Analysis (CFA) model: a Principal Component Analysis (PCA) was used to identify the latent structure, and to suggest an initial CFA model, which was further improved by removing items and refining the a priori food aggregation. Secondly, the validity of the model was checked using the validation sample (*n* = 571 remaining participants). Finally, the model was estimated using the whole sample and a bootstrap analysis was conducted to check the stability of the model loadings (Additional file 1: Figure S1). From this internal validation, food items were categorized as follow: “fruit and vegetables (FV)” (cucumber, grated carrots, tomatoes, cantaloupe, French beans, zucchini, stir fried vegetables, ratatouille, pear, orange, grape, kiwi, peach, Cronbach’s α = 0.75); “meat/fish/eggs (MFE)” (flank steak, lamb chops, lean fish, salmon, omelette, fried eggs, Cronbach’s α = 0.57); “cheese” (Gruyère, Camembert, Roquefort, goat cheese, Cronbach’s α = 0.66); “dessert” (vanilla puff, caramel custard, strawberry ice cream popsicle, Cronbach’s α = 0.52). Liking for each food group was assessed by the mean score of child’s liking of all of the food items included in that food group. The external validity and rest-retest reliability analyses, conducted among same age children (*n* = 19) confirmed that this tool was suitable for 5y children (data not shown).

### Sample selection

From the 2002 mothers recruited into the EDEN study, 95 were excluded for following reasons: withdrawal from the study, lost to follow up, miscarriage, in-utero death, moved out, abortions, missing birth weight. From the 1899 newborns, 1255 children were followed-up to 5y of age. Among them, 1147 participated in the food liking test. We excluded 5 children due to understanding difficulties (*n* = 4), or age under 5y (*n* = 1). Thus, our final sample included 1142 children. Compared with the 860 children not studied, the 1142 included were breastfed longer (3.3 ± 3.7 vs 3.0 ± 3.5 months, *p* =0.04), had an older mother (30 ± 5y vs 29 ± 5y, *p* <0.0001), had more often a non-smoker mother during pregnancy (79 vs 66 % of non-smokers, *p <* 0.0001), a mother with a higher educational level (58 vs 45 % with an university degree, *p* < 0.0001) and were more frequently recruited in Poitiers (56 vs 38 %, *p* < 0.0001) than in Nancy. There was no difference in newborn characteristics (gender: *p* =0.26; gestational age: *p* =0.22; birth weight: *p* =0.62; preterm birth: *p* =0.99).

### Statistical analysis

The associations between food liking and its early factors were estimated using a Structural Equation Modeling (SEM) approach to test theoretical models describing their relationships (Additional file 2: Figure S2). In our final SEM models, we keep only significant associations (*p* < 0.05), which were reported through arrows in our figures. To reduce the dimensionality of the data, we grouped highly correlated variables into latent variables as follow: “child’s fruit and vegetables intake” measured with fruit intake and vegetables intake (Cronbach’s α = 0.58), “maternal fruit and vegetables intake” measured with fruit, raw vegetables and cooked vegetables intakes (Cronbach’s α = 0.70), “maternal meat, fish and eggs intake” measured with meat, fish and eggs intakes (Cronbach’s α = 0.36). Considering items of their respective scale, Child Control (Cronbach’s α = 0.48), Restriction for Weight (Cronbach’s α = 0.74) and food neophobia at 1y (Cronbach’s α = 0.61), 2y (Cronbach’s α = 0.91), 3y (Cronbach’s α = 0.92) and 4y (Cronbach’s α = 0.83) were considered as latent variables. In our results, standardized parameter estimates were presented to remove scaling effects and allow comparisons between parameters in the model. Four fit indexes were used to judge the fit of each model: the Standardized Root Mean Square Residual (SRMSR), the Comparative Fit Index (CFI), the Root Mean Square Error of Approximation (RMSEA) and the Adjusted Goodness-of-fit index (AGFI). To indicate a good fit, SRMSR and RMSEA should be equal to or lower than 0.05 while CFI and AGFI should be equal to or lower than 0.90 [27]. To handle missing data in our SEM models, an incomplete-data maximum likelihood estimation, named “full-information maximum likelihood” (FIML), was applied. A similar structural equation model, presented in the appendix, was proposed for all food groups. We performed a sensitivity analysis among children without missing data (complete case analysis). All analyses used SAS (version 9.3, 2011, SAS Institute, Inc). For Structural Equation Modelling, we used PROC CALIS procedure and LINEQS modelling language.

## Results

The characteristics of study population are presented in Table 1. At 2y, restriction for weight and child control (1–5 scales) had mean scores of 1.7 and 2.3, respectively. The mean score of food neophobia (1–5 scales) was around 2 from 2 to 4 y. At 3y, the daily frequency of intake was 1.1 for FV, 1.2 for MFE, 2.5 for dairy products and 1.3 for desserts. At 5y, the food liking scores were 6.4 for both FV and MFE, 4.0 for cheese and 6.7 for desserts.

**Table 1:** Population characteristics at baseline

	Mean ± sd or *n* (%)
Number of mother/child pairs	1142
Maternal characteristics	
Age at delivery (years)	30 ± 5
Pre-pregnancy BMI (kg/m^2^)	23.4 ± 4.6
From Poitiers recruitment centre (%)	638 (56)
University degree (%)	658 (58)
Newborn characteristics	
Boys (%)	612 (54)
Gestational age (weeks)	39.3 ± 1.7
Birthweight (kg)	3.3 ± 0.5
Any breastfeeding duration (months)	3.3 ± 3.7
Age of complementary food introduction (months)	4.5 ± 1.6
Maternal food intake before pregnancy	
Fruit and vegetables (times/day)	3.4 ± 2.7
Meat, fish and eggs (times/day)	1.0 ± 0.3
Cakes (times/day)	0.6 ± 0.6
Cheese (times/day)	0.7 ± 0.3
Parental feeding practices at 2y (score ranked from 1 to 5)	
Restriction for weight	1.7 ± 0.6
Child control	2.3 ± 0.7
Average food neophobia between 1 to 4y (score ranked from 1 to 4)	2.1 ± 0.6
Food neophobia score at 1y	1.6 ± 0.6
Food neophobia score at 2y	2.0 ± 0.9
Food neophobia score at 3y	2.1 ± 0.8
Food neophobia score at 4y	2.1 ± 0.8
Child’s food intake at 3y	
Fruit and vegetables (times/day)	1.1 ± 0.6
Meat, fish, eggs (times/day)	1.2 ± 0.4
Dairy products (times/day)	2.5 ± 0.5
Dessert (times/day)	1.3 ± 0.3
Child’s food liking at 5y (score ranked from 0 to 10)	
Fruit and vegetables	6.4 ± 2.0
Meat, fish and eggs	6.4 ± 2.3
Cheese	4.0 ± 4.2
Dessert	6.7 ± 2.9

### Fruit and vegetables liking

**Fig. 1:**
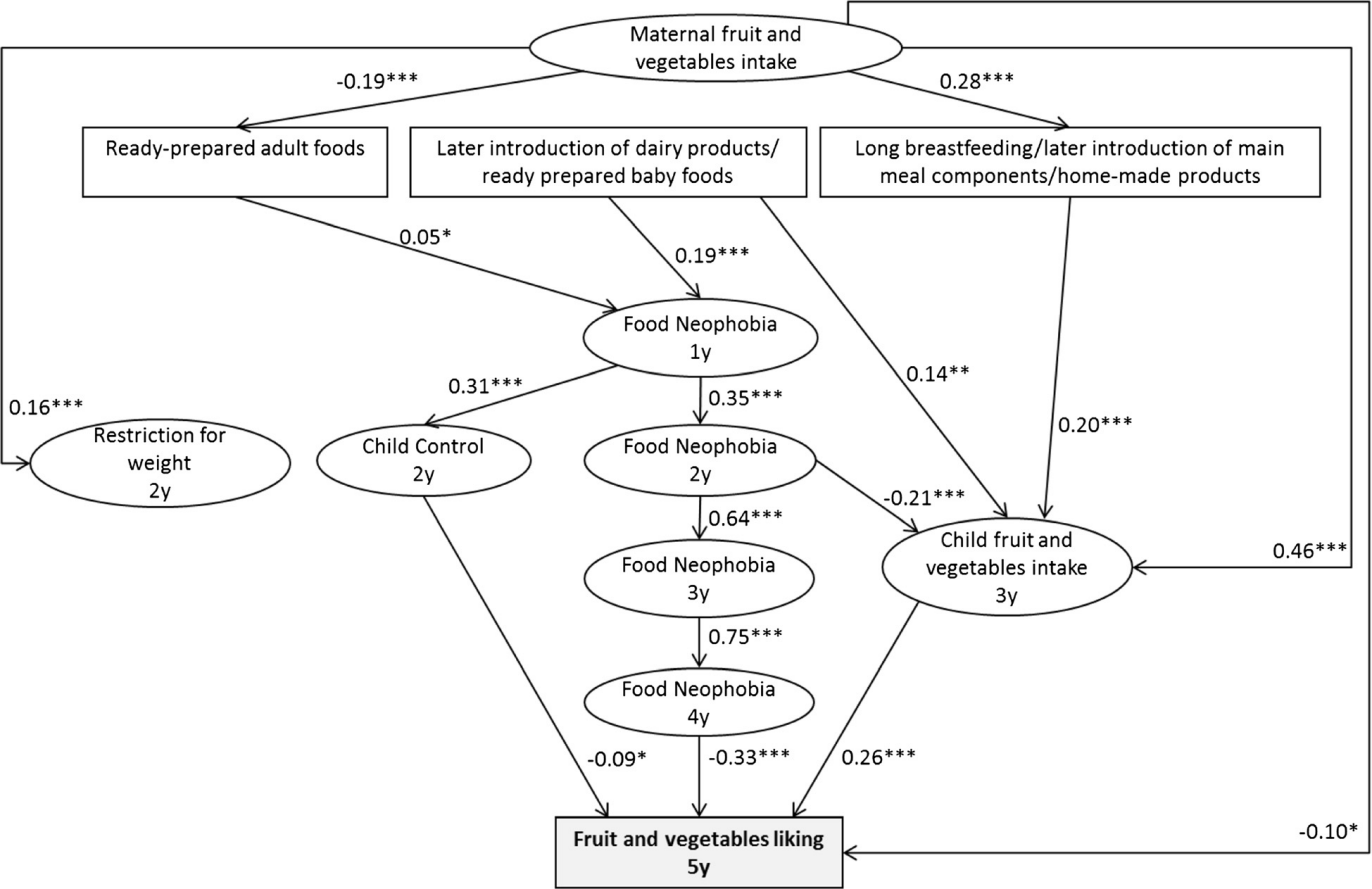
Early factors related to child’s liking of fruit and vegetables at 5y (*n* = 1142). Path coefficients in the model can be interpreted as standardized regression weights. Latent variables are presented in ovals and observed variables are presented in rectangles. Significance values: *p < 0.05, **p < 0.01, ***p < 0.001. Fit indexes for this model were SRMSR = 0.04, AGFI = 0.98, RMSEA = 0.03, CFI = 0.97. Infant feeding patterns were previously identified from breastfeeding duration, age of introduction to 14 complementary foods, type of food used at 12 month (home-made, ready-prepared baby food, ready prepared adult food) by principal component analysis [25]

FV intake at 3y (r = 0.26, *p* < 0.001), food neophobia at 4y (r = −0.33, *p* < 0.001), Child Control at 2y (r = −0.09, *p* < 0.05) and maternal FV intake before pregnancy (r = −0.10, *p* < 0.05) were directly related to FV liking at 5y (Fig. 1). Neither infant feeding patterns nor restriction for weight at 2y was related to FV liking at 5y.

### Meat, fish and eggs liking

**Fig. 2:**
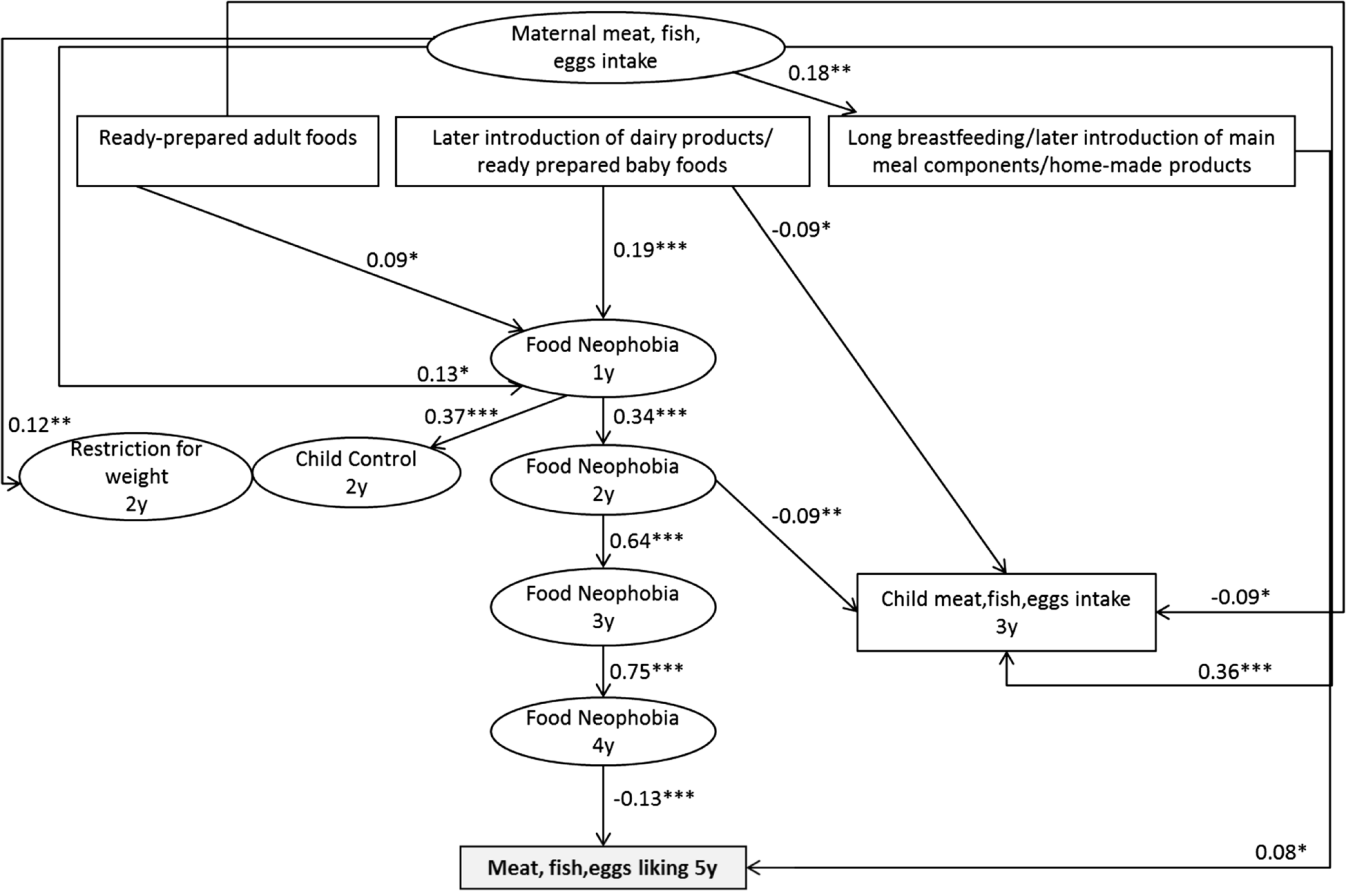
Early factors related to child’s liking of meat, fish and eggs at 5y (*n* = 1142). Path coefficients in the model can be interpreted as standardized regression weights. Latent variables are presented in ovals and observed variables are presented in rectangles. Significance values: *p < 0.05, **p < 0.01, ***p < 0.001. Fit indexes for this model were SRMSR = 0.04, AGFI = 0.99, RMSEA = 0.03, CFI = 0.98. Infant feeding patterns were previously identified from breastfeeding duration, age of introduction to 14 complementary foods, type of food used at 12 month (home-made, ready-prepared baby food, ready prepared adult food) by principal component analysis [25]

Food neophobia level at 4y (r = −0.13, *p* < 0.001) and infant feeding pattern-2 “Long breastfeeding, later introduction of main meal components and use of home-made products” were directly related to MFE liking at 5y (r = 0.08, *p* < 0.05) (Fig. 2). Maternal and child’s 3y MFE intake were not associated with MFE liking at 5y. No association was found with MFE liking and parental feeding practices at 2y (Child Control, Restriction for weight).

### Cheese liking

**Fig. 3:**
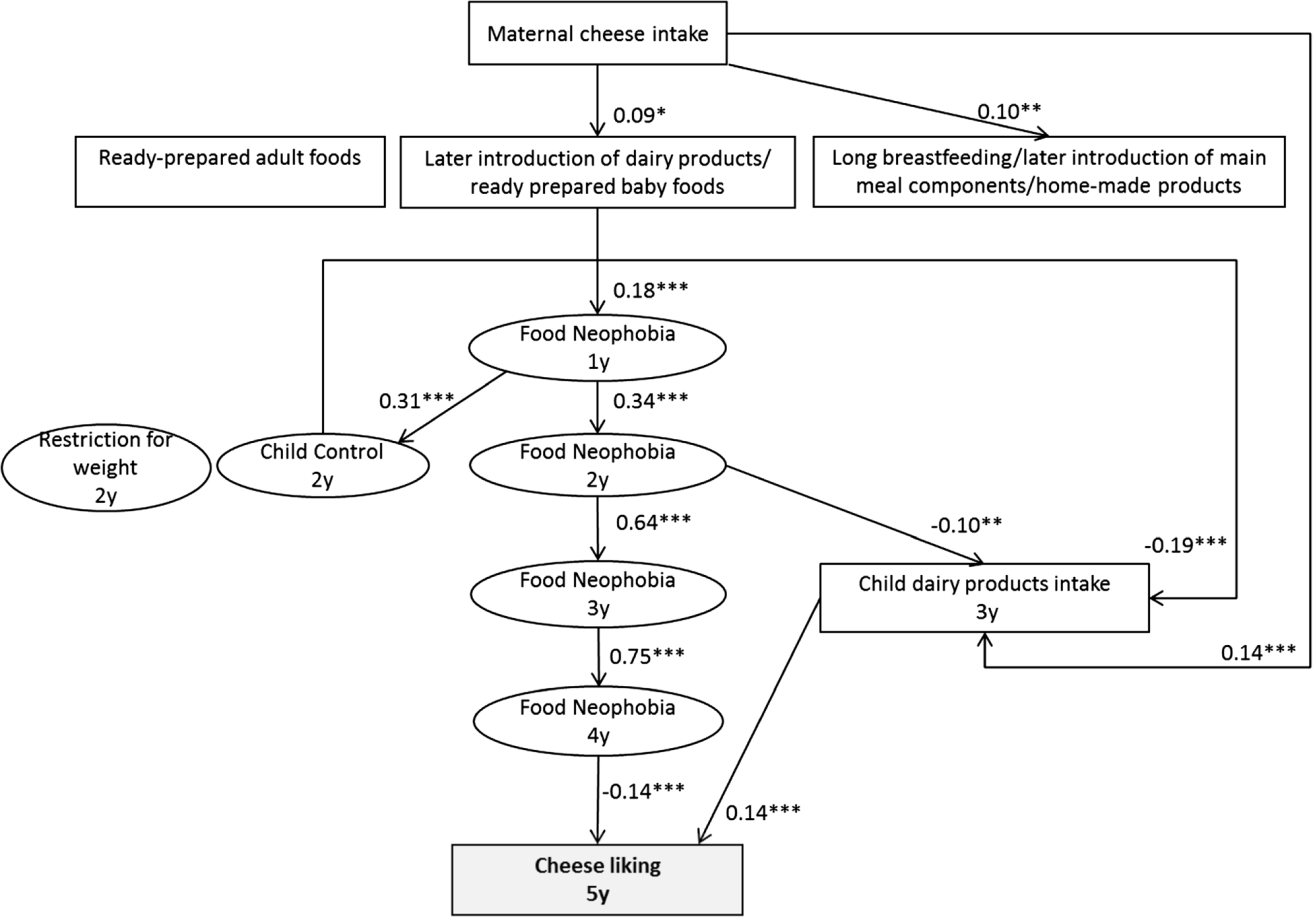
Early factors related to child’s liking of cheese at 5y (*n* = 1142). Path coefficients in the model can be interpreted as standardized regression weights. Latent variables are presented in ovals and observed variables are presented in rectangles. Significance values: *p < 0.05, **p < 0.01, ***p < 0.001. Fit indexes for this model were SRMSR = 0.04, AGFI = 0.99, RMSEA = 0.03, CFI = 0.98. Infant feeding patterns were previously identified from breastfeeding duration, age of introduction to 14 complementary foods, type of food used at 12 month (home-made, ready-prepared baby food, ready prepared adult food) by principal component analysis [25]

Dairy product intake at 3y (r = 0.14, *p* < 0.001) and child’s food neophobia (r = −0.14; *p* < 0.001) were directly related to Cheese liking at 5y (Fig. 3). Other factors such as maternal cheese intake, parental feeding practices at 2y and infant feeding patterns were not related to Cheese liking at 5y.

### Dessert liking

**Fig. 4:**
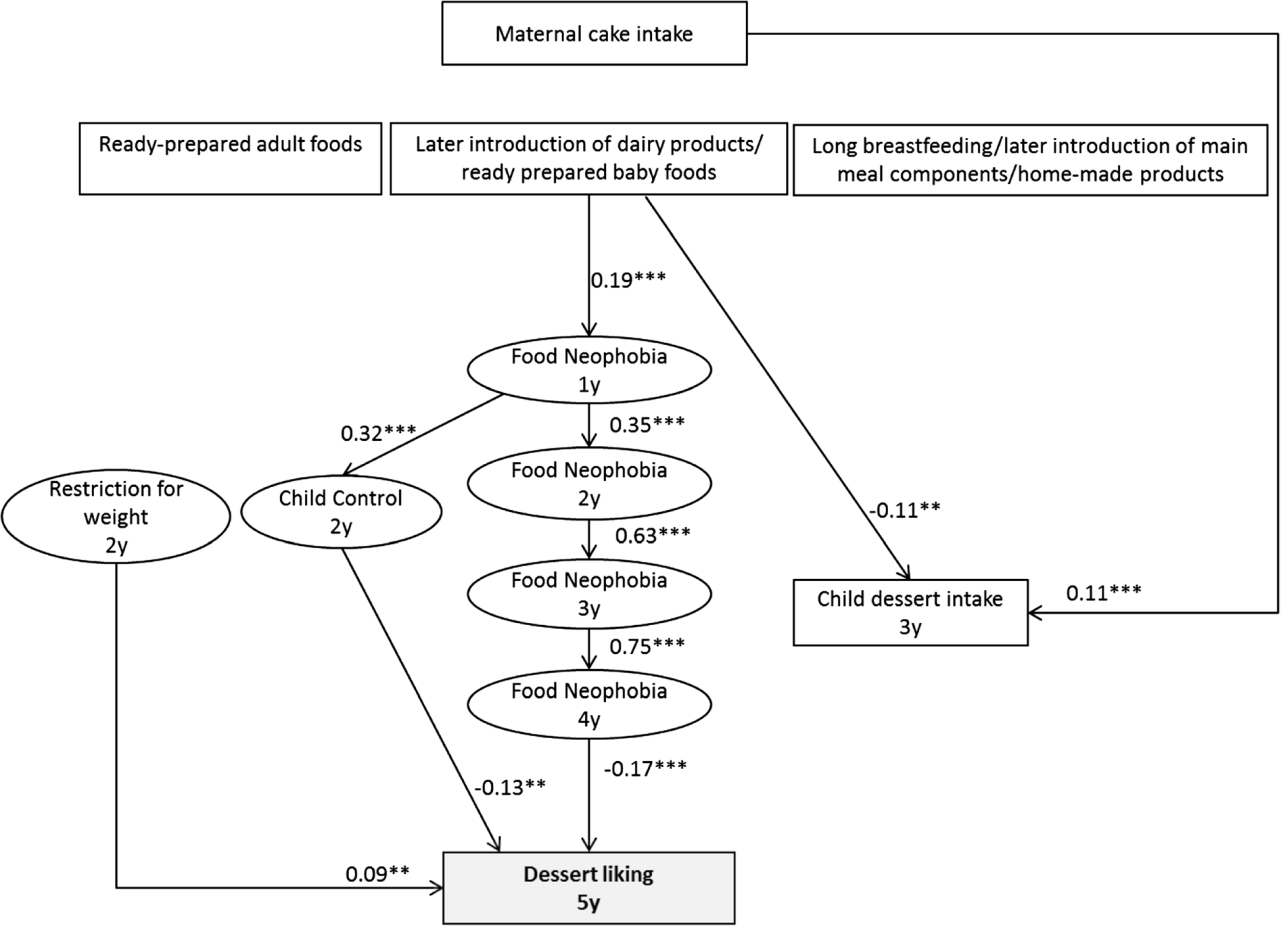
Early factors related to child’s liking of dessert at 5y (n = 1142). Path coefficients in the model can be interpreted as standardized regression weights. Latent variables are presented in ovals and observed variables are presented in rectangles. Significance values: *p < 0.05, **p < 0.01, ***p < 0.001. Fit indexes for this model were SRMSR = 0.04, AGFI = 0.99, RMSEA = 0.03, CFI = 0.98. Infant feeding patterns were previously identified from breastfeeding duration, age of introduction to 14 complementary foods, type of food used at 12 month (home-made, ready-prepared baby food, ready prepared adult food) by principal component analysis [25]

Low food neophobia (r = −0.17, *p* < 0.001), low level of Child Control (r = −0.13, *p* < 0.01) and high level of Restriction For Weight (r = 0.09, *p* < 0.01) were related to higher Dessert liking at 5y (Fig. 4). Neither maternal intake nor child’s dessert intake at 3y was related to Dessert liking at 5y. No association was found with infant feeding patterns and Dessert liking at 5y.

Results from the complete case analysis (data not shown) were similar to those presented above.

## Discussion

With a longitudinal design, we showed that the child’s food neophobia assessed at 4y was associated with child’s liking for all food groups at 5y, whereas the potential influence of other early factors depended on the food group considered. Our study is the first to use a face-to-face interview suitable for young children for assessing child’s food liking among a large sample size of children.

Food neophobia was negatively associated with food liking in children. As young children have to face a wide range of novel foods during the first few years of life, food neophobia may be an adaptive behavior to protect them from unfamiliar and potentially unsafe food [28]. Among the studies that have examined the association between food neophobia and food liking, most of them found an association with fruit and vegetables, either with intake [7, 29–31] or with liking [9, 11], and with liking meats [29, 31] but not with non-core food liking [11]. Similarly with our findings, Russell et al. found that food neophobia was negatively associated with both liking fruit and vegetables and liking meats, but also to a lesser extent with liking cereals, dairy products and extra foods (biscuits, pies, candy…) [10]. Experimental studies have demonstrated that frequent exposition to a food or a flavor may reduce food neophobia [28] and increase food liking during childhood [6, 32–34]. Birch et al. [35] have shown that repeated exposure to ready prepared baby food leads to a higher intake of the same or similar baby foods, but does not increase the intake of the home-prepared foods. In line with these findings, we found that the infant feeding pattern “later introduction of dairy products and the use of ready prepared baby foods” was positively related to food neophobia at 1y, but the other infant feeding patterns studied were not associated with food neophobia. One can argue that exposure to more sensory food as in home prepared food compared to ready prepared food could lead to lower neophobic reactions in children.

From our findings, child’s fruit and vegetables and dairy intakes at 3y were associated with fruit and vegetables and cheese liking at 5y, respectively. However, neither “meat, fish and eggs” nor dessert intakes at 3y were related to liking for the corresponding food group, potentially due to a lack of variability in child intakes. While sweet taste liking is innate, fruit and vegetables contain both bitter and sour tastes which have been shown to be rejected during the first months [36, 37], and cheese contains butyric acid and/or dimethyl disulphide with an unpleasant smell [38] and an acrid taste [39, 40]. Consequently, vegetables and cheese may need to be presented frequently to children before they accept and like them [41, 42]. Several studies have considered food liking as a determinant of food intake, but most of them involved pre-adolescents.

To our knowledge, there is only one study on the association between food liking and food intake among preschool children [43], showing that child’s fruit and vegetables intake was associated with fruit and vegetables liking. In this cross-sectional study, both child’s liking and child’s intake were reported by the mothers, increasing the risk of social desirability bias. As both liking and intake were assessed at the same period, it was not possible to examine the temporality of the association. Although food intake is obviously linked with what parents offer, food liking may be a better reflect of child’s future food choice without parental constraints.

From previous studies, the main predictor of child’s fruit and vegetables intake identified is maternal fruit and vegetables intake [7, 30, 31, 43, 44]: the more the mothers consumed fruit and vegetables, the more their child consumed fruit and vegetables. Consistent with these findings, we found that maternal intake was associated positively with child’s intakes at 3y for all food groups, especially fruit and vegetables and meat, fish and eggs. To explain this relation, many hypotheses have been explored, such as parental modeling [45], but also food availability and early exposure that may influence later eating habits and food liking. Previous studies have shown that maternal liking was related with their child’s liking at 2y [11], and at 8y [9]. Even if a genetic link was not demonstrated between child and parental food liking, the heritability of food neophobia was estimated between 67 and 78 % [46, 47].

Parental feeding practices may also have a key role on the development of child’s food liking. However, most studies have focused on their influence on child’s intake, often reported by parents, rather than on self-declared child’s liking for foods. In our study, we found that Child Control (“parents allow the child to control of his/her eating behaviors”) was negatively related to dairy products intake at 3y and to liking dessert or fruit and vegetables at 5y. The association may be explained since children with a higher score in child control can get more easily dessert or can refuse more easily fruit and vegetables, they may have different liking for those foods compared with children with a lower score in Child control, that is to say, compared with children who are less free in deciding what they eat. Hennessy et al. [16] have shown that being highly responsive to child’s requests and setting few demands on them may lead the child to consume higher quantities of low nutrient energy foods but not higher amounts of healthy foods. Similarly, Vereecken et al. [48] have highlighted that a parental “permissiveness” feeding style was related negatively with fruit and vegetables intake and positively with soft drinks and sweets intake. The influence of parental restriction of a child’s intake on their eating behavior is better established. Birch et al. have shown in a longitudinal study, that early restrictive feeding practice on snack foods may lead a child to eat more of these foods in the absence of hunger than a child who was not exposed to restrictive feeding practices [49]. In experimental studies, forbidding certain foods, especially sweet foods, may lead children to be more attracted to these foods, and consequently to have higher intake when these foods are freely available compared with children who were not restricted [14, 15, 49, 50]. Consistent with these results, we found that the Restriction for Weight Control score was positively related to dessert liking at 5y, but not to the other food groups.

In our study, child’s food liking was assessed by a face-to-face interview with a food liking test, in contrast to all other studies on this topic that used parental reports. The main issue with an indirect assessment of child’s food liking with a questionnaire completed by their mothers is that mothers may be influenced by their own preferences in reporting their child’s [51]. In addition, as children were followed from birth to 5y, we were able to study temporality of these variables. Our study has however some limits. Because no validated tools were available to evaluate food neophobia at the beginning of the study for infants and toddler, we decided to create ad hoc items (for the need of the study). As for most of the studies on this topic [9, 11], the present study included families that were more socio-economically advantaged with a higher education level than families not studied and thus the results may not be applicable to family of lower socioeconomic status. It cannot be ruled out that child’s liking report may slightly vary from 1 day to day. As child was considered to be able at this age to report accurately he/her dietary intake [52], we presumed the same for food liking. The lack of association between child’s food liking at 5y and food dietary intake at 3y for several food groups could be partially explained by the lack of correspondence between items from the food liking test and the FFQ. Finally, the FFQ used in the present study was set to cover the whole diet but it hasn’t been validated.

## Conclusion

Our study is unique, as we were able to consider both the complex and temporal relationships between the determinants of food liking assessed directly with a face-to-face interview with the child. Our results suggest that food neophobia is strongly and positively associated with liking for all food groups among children, especially for fruit and vegetables. In contrast, food liking at 5y was not associated directly with feeding practices in the first year of life. Nevertheless, fruit and vegetables and cheese liking at 5y were related to their respective intake frequency at 3y. These results suggest that compared with other food groups, fruit and vegetables and cheese should be presented earlier and more frequently to children to encourage their liking and consumption.

## Additional files

Additional file 1: Figure S1. Confirmatory Factor Analysis (CFA) model with the total sample (*n* = 1142). (DOCX 121 kb) Additional file 2: Figure S2. Theoretical model. (DOCX 108 kb)

## Abbreviations

EDEN: Etude des Déterminants pre- et post-natals de la santé de l’ENfant
FV: fruit and vegetables
MFE: meat, fish and eggs
SEM: structural equations modelling
