# Supplementary material for: Early determinants of food liking among 5y-old children: a longitudinal study from the EDEN mother-child cohort
Source: Int J Behav Nutr Phys Act. 2016 Feb 15;13:20. doi: 10.1186/s12966-016-0342-5 (PMC4753648; doi:10.1186/s12966-016-0342-5)
Supplement: Additional file 1: Figure S1. — Confirmatory Factor Analysis (CFA) model with the total sample (n = 1142). (DOCX 121 kb) [file 12966_2016_342_MOESM1_ESM.docx]

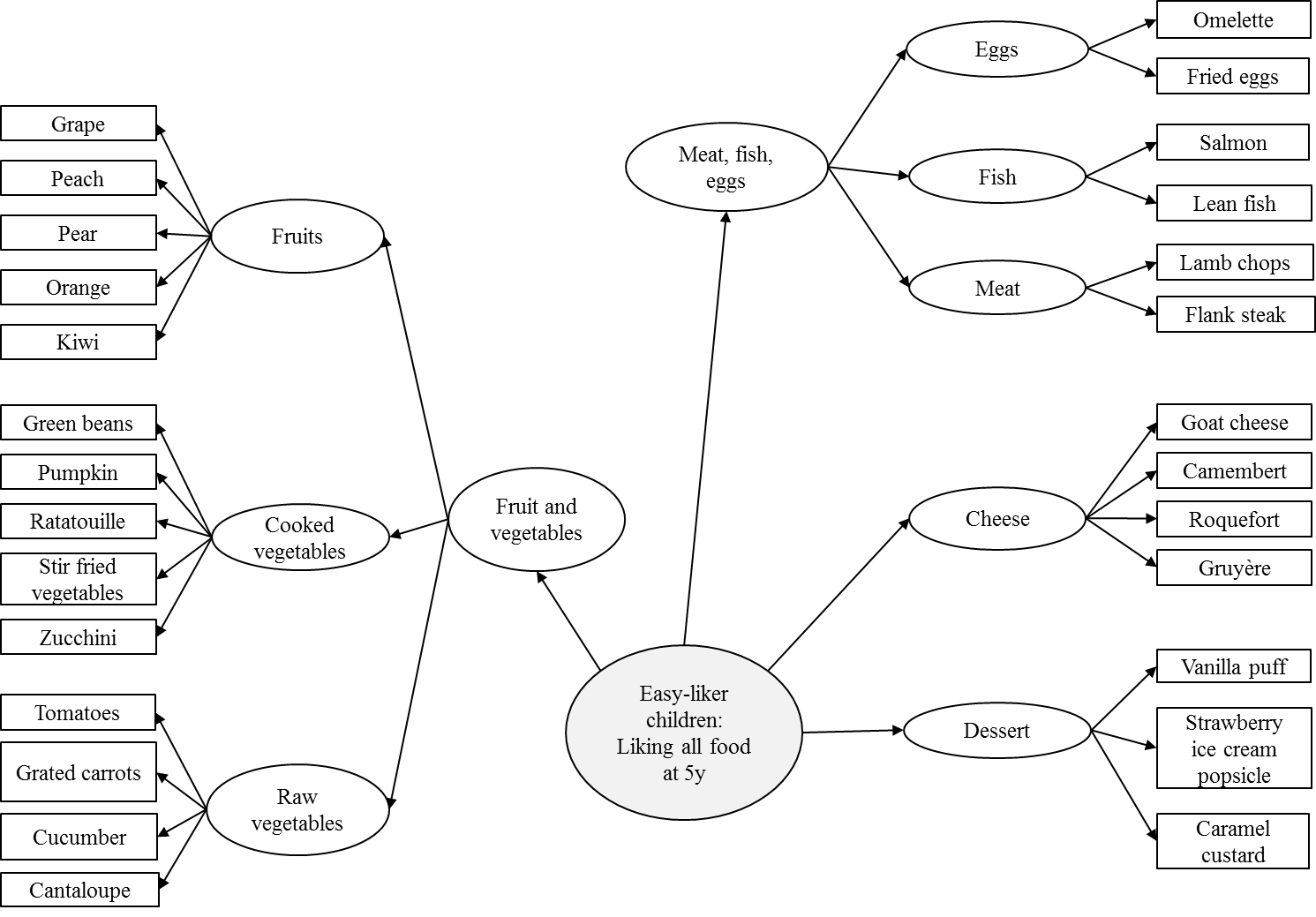


Supplemental Figure 1 Confirmatory Factor Analysis (CFA) model with the total sample (n=1142).

*Figure legend*

Latent variables are presented in ovals and observed variables are presented in rectangles. Fit indexes for this model were SRMSR=0.04, RMSEA= 0.03, CFI=0.93.
