# Supplementary material for: Early determinants of food liking among 5y-old children: a longitudinal study from the EDEN mother-child cohort
Source: Int J Behav Nutr Phys Act. 2016 Feb 15;13:20. doi: 10.1186/s12966-016-0342-5 (PMC4753648; doi:10.1186/s12966-016-0342-5)
Supplement: Additional file 2: Figure S2. — Theoretical model. (DOCX 108 kb) [file 12966_2016_342_MOESM2_ESM.docx]

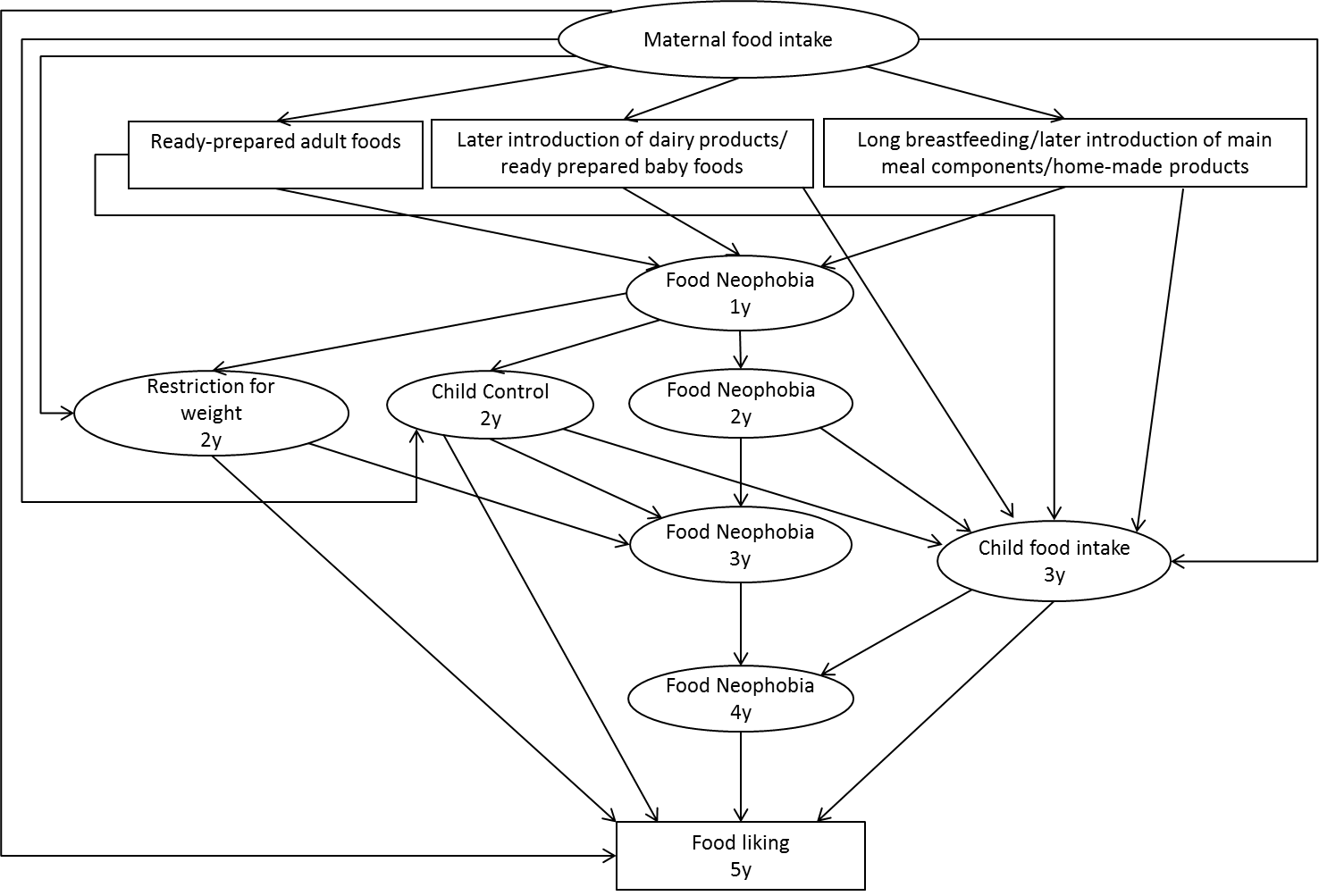


Supplemental Figure 2 Theoretical model

Latent variables are presented in ovals and observed variables are presented in rectangles.
